# The transmembrane transporter domain of glutamate transporters is a process tip localizer

**DOI:** 10.1038/srep09032

**Published:** 2015-03-12

**Authors:** Mariko Kato Hayashi, Masato Yasui

**Affiliations:** 1 Department of Pharmacology, Keio University School of Medicine, Shinjuku, Tokyo 160-8582, Japan

## Abstract

Glutamate transporters in the central nervous system remove glutamate released from neurons to terminate the signal. These transporters localize to astrocyte process tips approaching neuronal synapses. The mechanisms underlying the localization of glutamate transporters to these processes, however, are not known. In this study, we demonstrate that the trimeric transmembrane transporter domain fragment of glutamate transporters, lacking both N- and C-terminal cytoplasmic regions, localized to filopodia tips. This is a common property of trimeric transporters including a neutral amino acid transporter ASCT1. Astrocyte specific proteins are not required for the filopodia tip localization. An extracellular loop at the centre of the 4^th^ transmembrane helices, unique for metazoans, is required for the localization. Moreover, a C186S mutation at the 4^th^ transmembrane region of EAAT1, found in episodic ataxia patients, significantly decreased its process tip localization. The transmembrane transporter domain fragments of glutamate transporters also localized to astrocyte process tips in cultured hippocampal slice. These results indicate that the transmembrane transporter domain of glutamate transporters have an additional function as a sorting signal to process tips.

Glutamate is a major excitatory neurotransmitter of the vertebrate central nervous system^1^. Glutamate released from presynaptic terminus activates ionotropic or metabotropic glutamate receptors of postsynaptic neurons. Activation of ionotropic glutamate receptors depolarizes the postsynaptic membranes, and calcium permeable ionotropic glutamate receptors or Gq-coupled metabotropic glutamate receptors raise cytoplasmic calcium concentration^2^. After successful activation of postsynaptic neurons, the glutamate must be removed from the synaptic cleft to terminate the signal and prepare for another cycle of glutamate release. Furthermore, activation of calcium-signalling cascade triggered by excessive stimulation of glutamate receptors leads to neuronal apoptosis, which is a cause of poor prognosis of brain injury, ischemia or neurodegenerative diseases^3^. For these reasons, both neurons and astrocytes have Na^+^-dependent glutamate transporters that mediate rapid uptake of glutamate from extracellular fluids^4^. As a Na^+^-glutamate symporter, these glutamate transporters use electrochemical gradient of sodium ion across the plasma membrane to transport extracellular glutamate to cytoplasm.

Mammals have five subtypes of glutamate transporters. Two major glutamate transporter subtypes in brain are primarily expressed in astrocytes^5^: EAAT1 (excitatory amino acid transporter 1)/GLAST-1 (glutamate-aspartate transporter) and EAAT2/GLT-1 (glial glutamate transporter 1). EAAT2 is primarily responsible for taking up released glutamate in the central nervous system^6^.

These glutamate transporters belong to solute carrier family 1 (SLC1) of transporters. SLC1 transporters are secondary active transporters which use electrochemical gradient of sodium to transport substrates. SLC1 family transporters including glutamate transporters have 2 helical hairpins for substrate binding, and form a trimer with a concave surface at the centre of the extracellular face^7^.

As their name implies, astrocytes have several stem branches, each containing thousands of finely branched processes that extend from the stems to the surface of neurons^8^,^9^,^10^. Glutamate transporters are enriched in tips of these processes at sites of contact with neurons^11^,^12^,^13^,^14^,^15^. However, the molecular mechanisms responsible for the localization of transporters in these processes are unknown.

In this study, we report that the transmembrane SLC1 transporter domain of glutamate transporters mediates filopodia tip localization in astrocytes in primary culture. Their cytoplasmic tails known as binding sites of cytosolic scaffolding proteins are not necessary. The transmembrane transporter domain fragments also localized to filopodia tips of COS-7 cells, indicating astrocyte specific protein is not required for the localization. Our studies indicate that the function of SLC1 transporter domain is not limited to transporting substrates, and it acts as a sorting signal to process tips.

## Results

### Differential localization of glutamate transporters

**Figure 1:**
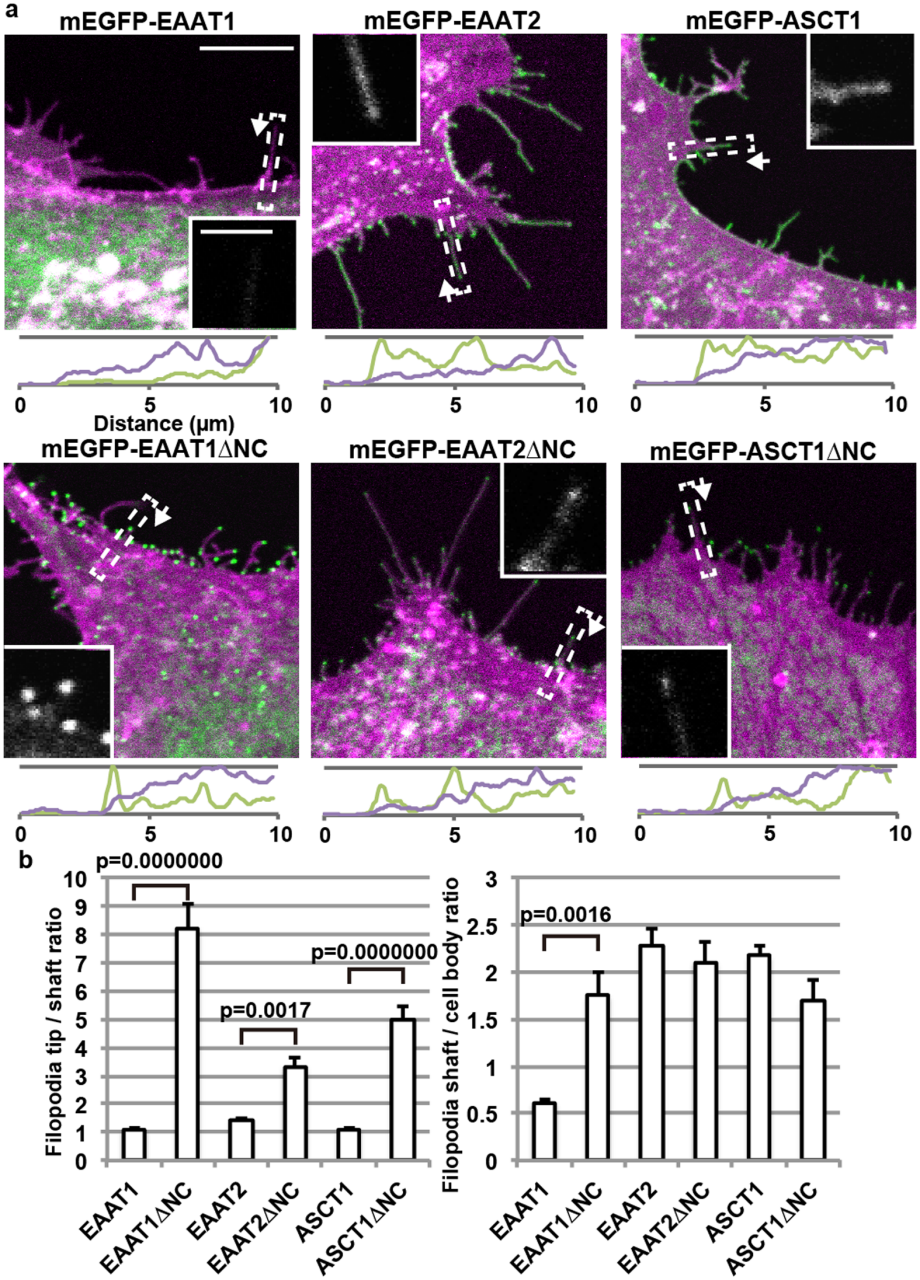
Filopodia localization of SLC1 transporters in primary cultured astrocytes. (a) Top: Primary cultured astrocytes were co-transfected with recombinant adenoviruses encoding mEGFP-labelled transporters (green) or Lck-mCherry as a plasma membrane marker (magenta). Scale bar, 10 μm. Bottom: mEGFP (green) and mCherry (magenta) signal intensity scanned along filopodia indicated by boxes in the top panels. Representative filopodia were scanned from the tip (left) to the soma (right) as indicated by arrows. Vertical axis: arbitrary units for fluorescence intensity. Inset: mEGFP-channel images of the filopodia used for the signal intensity scan analysis. Scale bar, 2 μm. (b) Left: Filopodia tip localization of the transporters, calculated as ratios of the average signal intensity within 0.5 μm from a tip and the average signal intensity of the following 0.5 μm (shaft) of the filopodia. Right: Filopodia localization of the transporters, calculated as ratios of the average signal intensities within 1 μm length of a filopodia next to the cell body, and the following 1 μm length within the cell body, normalized by the signal intensity of Lck-mCherry. 71–143 cells for each construct were analysed in a blinded manner. Data are presented as mean ± s.e.m., with p values of Tukey-Kramer test.

First, we studied whether the localization of two major glutamate transporters in astrocytes, EAAT1 and EAAT2, is differentially regulated. We expressed EAAT1 or EAAT2 with mEGFP fused to the N-terminus, mEGFP-EAAT1 or mEGFP-EAAT2, in astrocytes in primary culture in combination with the plasma membrane marker Lck-mCherry^16^ using recombinant adenoviruses. EAAT1 and EAAT2 transporters showed a marked difference in localization. mEGFP-EAAT1 localized to both plasma membrane and endoplasmic reticulum (ER), and it was rather excluded from filopodia, with the 0.6 fold density compared to the soma (Fig. 1). In contrast, mEGFP-EAAT2 localized primarily to the plasma membrane, with stronger localization to filopodia than to soma by 2.3 fold. These results are consistent with previously reported localization for both endogenous EAAT2 and exogenously introduced GFP-EAAT2^17^,^18^.

### Transmembrane transporter domain of glutamate transporters is responsible for process tip localization

Next, to figure out which part of EAAT2 mediates the filopodial localization, we studied effects of removing its N- and C-terminal cytoplasmic tails. We truncated the C terminal cytoplasmic tail according to a previous study^19^, at a site that preserves glutamate uptake activity. Several cellular proteins bind to the cytoplasmic tails of EAAT2 that may potentially regulate its localization. For example, PDZ domain proteins PSD-95 and PICK1 bind to the C-terminus of GLT-1b, an alternatively spliced form at the C-terminus of EAAT2^20^,^21^. Another PDZ domain protein, MAGI, binds to internal sequences of EAAT2/GLT-1b C-terminal cytoplasmic tail^22^. In addition, a LIM domain protein Ajuba binds to the N-terminal cytoplasmic tail of EAAT2 to facilitate its plasma membrane localization^23^.

Contrary to our expectations, after the removal of both cytoplasmic C- and N-termini (EAAT2ΔNC), localization of EAAT2 to filopodia tips became stronger, 3.3 times higher density at the tip, with some transporters retained within the ER (Fig. 1). Surprisingly, EAAT1, which does not localize to filopodia as a full-length protein, also localized to filopodia tips with 8.2 fold higher density than the shaft, when both N- and C-termini were removed (EAAT1ΔNC). These results suggest that the filopodia tip localization is a common property of the transmembrane transporter domain of glutamate transporters.

### Filopodia tip localization of SLC1 family transporters

EAAT1 and EAAT2 are solute carrier family (SLC1) transporters, which form bowl-shaped trimers. In mammals, there are two groups of proteins classified as SLC1 family transporters: glutamate transporters EAAT1-5, and neutral amino acid (alanine/serine/cysteine/threonine) transporters ASCT1-2. To determine if filopodial localization is a property specific for glutamate transporters or it also applies to ASCTs, we studied localization of ASCT1 in astrocytes in primary cultures. Full-length ASCT1 showed 2.2 fold stronger localization to the entire length of filopodia than the soma, while its localization to filopodia tips are not stronger (1.1 fold) compared to the shaft of filopodia (Fig. 1). In contrast, transmembrane transporter domain fragment of ASCT1 (ASCT1ΔNC) showed significantly stronger localization to filopodia tips, by 5.5 fold. These results indicate that the filopodia tip localization should be considered as a common property of the transmembrane domain of mammalian SLC1 transporters.

**Figure 2:**
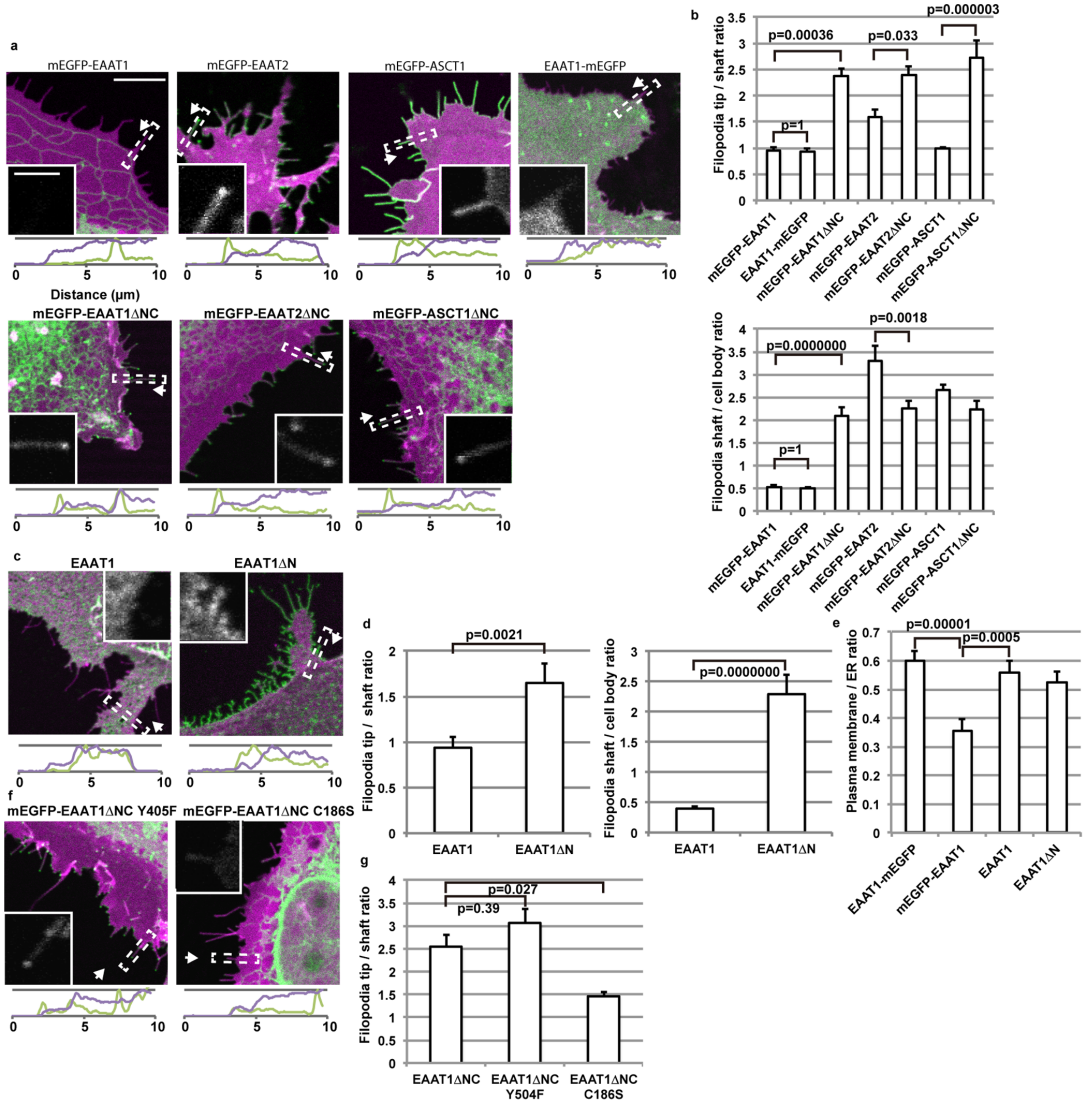
Filopodia localization of transporter mutants in COS-7 cells. Images are analysed and shown as described in Figure 1. (a) COS-7 cells expressing mEGFP-labelled transporters (green) or the plasma membrane marker Lck-mCherry (magenta). Scale bar, 10 μm. Inset: mEGFP-channel images of the filopodia used for the signal intensity scan. Scale bar, 2 μm. (b) Top: Filopodia tip localization of the transporters. Bottom: Filopodia localization of the transporters. 40–94 cells for each construct were analysed. Data are presented as mean ± s.e.m., with p values of Tukey-Kramer test. (c) COS-7 cells expressing EAAT1 or its N terminal deletion mutant (green) or Lck-mCherry (magenta). EAAT1 was immunostained with antibody raised against the C terminus peptide of EAAT1. (d) EAAT1 localization to filopodia tips and to filopodia body. 30 cells for each construct were analysed. Data are presented as mean ± s.e.m., with p values of Student's T test. (e) Plasma membrane – ER localization of EAAT1, analysed by calculating ratios of the signal intensities at plasma membrane and ER nearby. 30 cells for each construct were analysed. Data are presented as mean ± s.e.m., with p values of Tukey-Kramer test. (f) COS-7 cells expressing mEGFP-labelled EAAT1ΔNC mutants (green) or Lck-mCherry (magenta). (g) Filopodia tip localization of mEGFP-EAAT1ΔNC mutants. 50–97 cells for each construct were analysed, and presented as mean ± s.e.m., with p values of Tukey-Kramer test.

To figure out if filopodial localization of the SLC1 transporters requires astrocyte- specific proteins, we expressed the above constructs in COS-7 cells. The transmembrane transporter domain fragments of EAAT1, EAAT2 and ASCT1 localized to filopodia tips, reproducing the results obtained with astrocytes (Fig. 2a, b). These results indicate that proteins specifically expressed in astrocytes are not needed for filopodia tip localization of transmembrane transporter domains of glutamate transporters. The removal of cytoplasmic tails of EAAT2 resulted in enhanced localization to filopodia tips and reduction of localization to filopodia shaft in COS-7 cells. This result indicates that localization of transporters to filopodia tips and shafts are differentially regulated.

It has been reported that endogenous EAAT1 localizes to cell surface and not to ER in astrocytes in primary culture^24^, and we also confirmed this localization using EAAT1 without tag expressed in COS-7 cells (Fig. 2c, d). To verify whether fusion of mEGFP at the N terminal cytoplasmic tail of EAAT1, where an RXR-type ER retention signal is observed at residues 23–25, affects its localization, we compared plasma membrane localization of EAAT1 with or without mEGFP (Fig. 2a–e). The tag-free EAAT1 and EAAT1 with mEGFP at the C terminus localized to plasma membrane, while EAAT1 with mEGFP at the N terminus was significantly retained in ER, probably by exposing the ER retention signal at the N terminal cytoplasmic tail. On the other hand, all of these intact constructs showed weaker signal at filopodia compared to the cell body, by 0.4–0.5 fold (Fig. 2c, d). Removal of entire N terminal cytoplasmic tail of EAAT1 (EAAT1ΔN) was required for its localization to filopodia (2.3 fold) and to their tips (1.6 fold) (Fig. 2c, d).

### Transporter activity is not required for process tip localization

To figure out if transporter activity is required for filopodia tip localization of glutamate transporters, we introduced mutations known to completely abolish the transporter activity of EAAT1^25^. The Y405F mutant of EAAT1ΔNC without transporter activity retained filopodia tip localization to a similar extent as EAAT1ΔNC without the mutation (Fig. 2f, g). This result indicates that transporter activity is not required for process tip localization of SLC1 transporters.

### An intra-bowl insertion of SLC1 transporters is required for the filopodia tip localization

**Figure 3:**
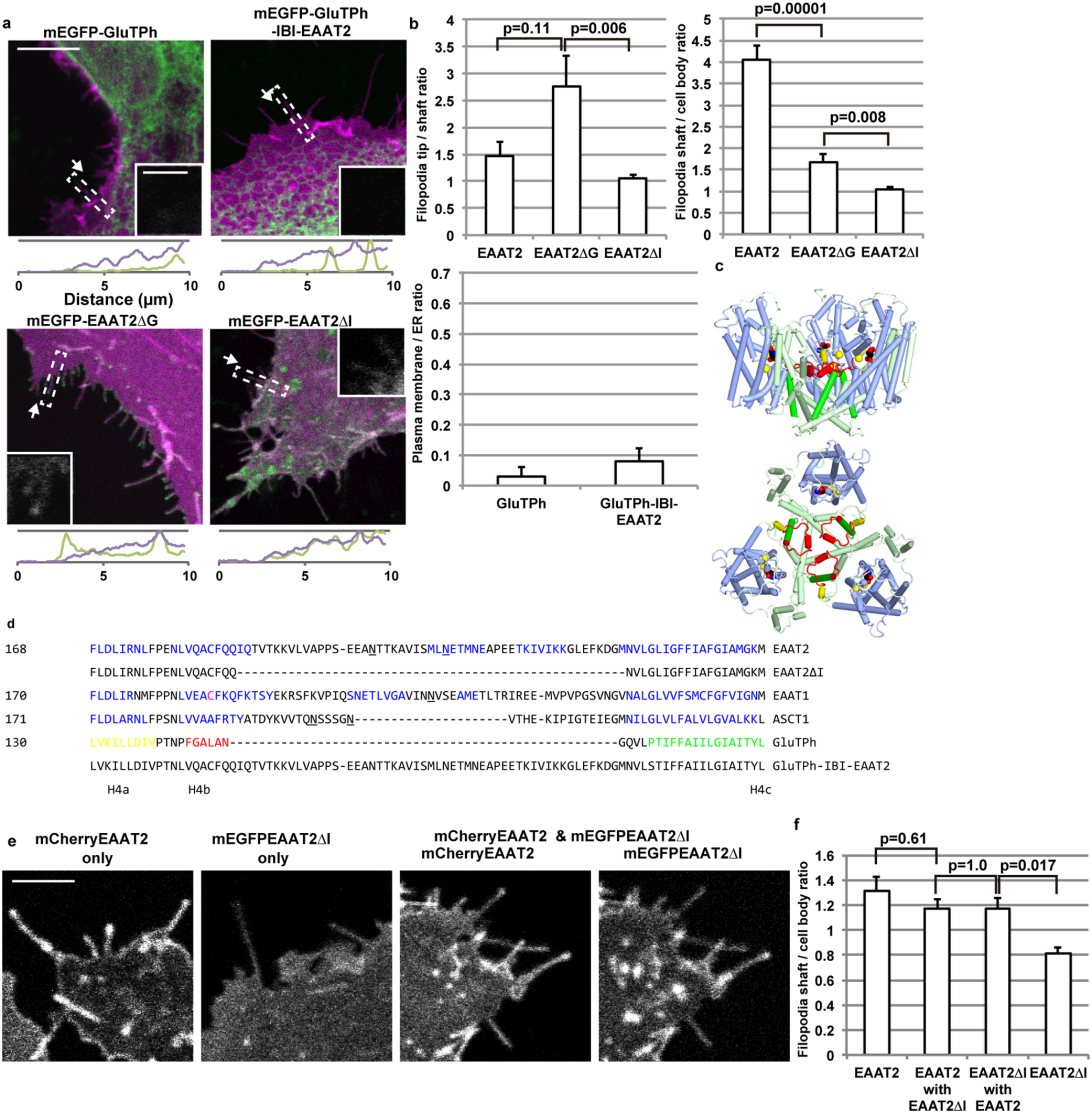
Requirement of intra-bowl insertion for filopodia tip localization. Images are analysed and shown as described in Figure 1. (a) mEGFP-fused GluTPh, GluTPh with the intra-bowl insertion of EAAT2, or EAAT2 mutants lacking the glycosylation sites (ΔG), or the intra-bowl insertion (ΔI) are expressed with Lck-mCherry in COS-7 cells. Scale bar, 10 μm. Inset, scale bar, 2 μm. (b) Left: Filopodia tip localization of the transporters. Right: Filopodia localization of the transporters. 50 cells for each construct were analysed, and presented as mean ± s.e.m., with p values of Tukey-Kramer test. Bottom: Plasma membrane – ER localization of various constructs of EAAT1. 30 cells for each construct were analysed, and presented as mean ± s.e.m. (c) Ribbon diagram of the crystal structure of trimeric GluTPh (PDBID: 2NWX). Yellow spheres represent sodium ions, red, black or blue spheres represent a substrate aspartate. Light green: trimerization subdomain, light blue: transport subdomain. Three helices of the 4^th^ transmembrane region, H4a, H4b and H4c are in yellow, red and green, respectively. The site of the intra-bowl insertion corresponds to H4b in red. (d) Amino acid sequence alignment using Clustal^44^. GluTPh sequences corresponding to H4a, H4b, H4c based on its crystal structure are labeled in yellow, red and green, respectively. Blue color indicates sequences predicted to be helical by a secondary-structure prediction program PredictProtein^45^. C186 of EAAT1 is in magenta. N-glycosylation sites are underlined. (e) COS-7 cells expressing mEGFP-EAAT2ΔI or mCherry-EAAT2 alone, or both mEGFP-EAAT2ΔI and mCherry-EAAT2. mEGFP channel and mCherry channel images are shown separately. Fluorescence intensities of these images are linearly adjusted for each so that average intensities of the background and the soma are same among these images. Scale bar, 5 μm. (f) Filopodia localization of the transporters. Note that the values are lower than (b), because normalization by Lck-mCherry signal intensity could not be applied. 30 cells for each construct were analysed. Data are presented as mean ± s.e.m., with p values of Tukey-Kramer test.

While the transmembrane transporter domains of mammalian SLC1 transporters localized to filopodia tips, an archaea glutamate transporter homologue, *Pyrococcus horikoshii* GluTPh, remained in ER and failed to localize to filopodia (Fig. 3a, b). Crystal structures of GluTPh show that it forms a bowl-shaped trimer^7^,^26^ (Fig. 3c). Comparison of the amino acid sequences of SLC1 transporters shows that metazoan SLC1 transporters have an insertion within the 4th transmembrane region consisting of 3 helices. The site of insertion is located at the centre of the extracellular concave surface of the bowl-shaped trimers, not far from their substrate-binding site.

All of these intra-bowl insertions of EAAT or ASCT are targets for N-glycosylation (Fig. 3d)^15^,^25^,^27^,^28^,^29^. We speculated that the glycosylation of this insert might be required for filopodia tip localization of mammalian SLC1 transporters. However, mutations introduced to the two N-glycosylation sites of EAAT2 (EAAT2ΔG) enhanced its localization to filopodia tips, from 1.5 fold to 2.8 fold higher density at tips compared to shafts, while reducing density at filopodia shafts compared to the cell body from 2.7 fold to 1.7 fold (Fig. 3a, b).

Next, we studied the effect of intra-bowl insertion removal. Most loops of the transmembrane transporter domain are just sufficiently long to connect between transmembrane regions. The only exception is the intra-bowl insertion unique for metazoans. We deleted the intra-bowl insertion of EAAT2 (EAAT2ΔI). The deletion mutants still localized to plasma membrane, but its localization to filopodia or to their tips were lost, to 1.1 fold signal intensity (Fig. 3a, b). These results indicate that the intra-bowl insertion is required for filopodia localization. To confirm that the loss of filopodia localization is not due to protein misfolding caused by the deletion, we co-expressed mCherry-labelled EAAT2 with mEGFP-labelled EAAT2ΔI. The co-expression of EAAT2 restored filopodia localization of EAAT2ΔI (Fig. 3e, f). This result implies that the deletion mutant correctly folds to form hetero-trimers with wild type EAAT2. It also indicates that three copies of the intra-bowl insertion or their trimer formation are not required for filopodia localization, but rather one or two copies within a single transporter trimer are sufficient.

To figure out if the intra-bowl insertions are sufficient for filopodia tip localization, we inserted the encoding sequences of the intra-bowl insertions of EAAT2 into mEGFP-GluTPh. However, EAAT2 intra-bowl insertion failed to confer plasma membrane localization or filopodia localization to GluTPh. The chimeric GluTPh construct was exclusively localized to ER, similar to the wild type GluTPh (Fig. 3a, b). These results indicate that the intra-bowl insertion is not sufficient for plasma membrane delivery or for filopodia localization. The insertion may be functional only as a part of the transporter domain cooperating with other parts of the transporter.

### Effect of C186S mutation of episodic ataxia patients

C186S mutation of EAAT1 was found in a family with episodic ataxia. This mutation reduced the glutamate uptake activity of EAAT1 by 18%^30^. C186 residue is localized at the second helix of the 4^th^ transmembrane region, near the N terminal of the intra-bowl insertion required for process tip localization. We speculated that the mutation might also affect the process tip localization of EAAT1. Since EAAT1 mostly localize to ER and not to filopodia tips in cultured cells, we used the transmembrane transporter domain fragment, EAAT1ΔNC, to evaluate the effect of the C186S mutation. The C186S mutation significantly reduced the extent of filopodia tip localization of EAAT1ΔNC from 2.5 fold to 1.5 fold (Fig. 2f, g). This result suggests that the failure of the C186S mutant to properly localize to process tips might be another cause of the cerebellar malfunction.

### Astrocyte process tip localization of glutamate transporters in organotypic hippocampal slice cultures

**Figure 4:**
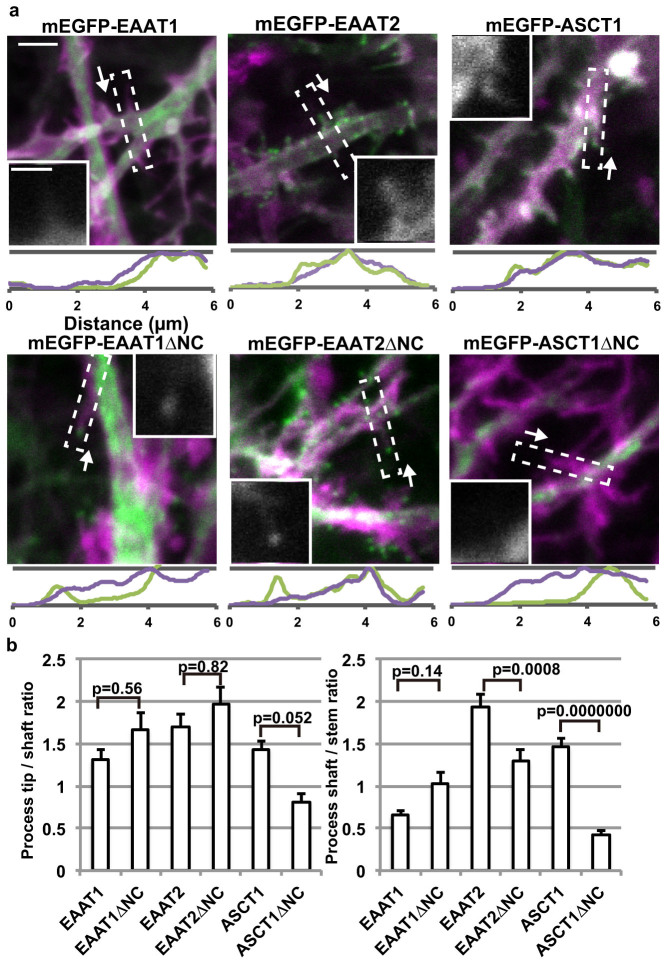
Localization of SLC1 transporters in astrocytes in hippocampal organotypic slice cultures. (a) Top: Images of astrocytes in organotypic hippocampal slice cultures co-transfected with recombinant adenoviruses encoding mEGFP-labelled transporters (green) or Lck-mCherry (magenta) at DIV 8–10. Summed stacks of a series of Z-section images taken within a range of 3–5 μm-thickness at 0.5 μm intervals are shown, with signal intensity linearly adjusted for each channel. Scale bar, 2 μm. Bottom: Signal intensities scanned along a process indicated by boxes in the top panel are shown below each microphotograph. Processes were scanned from the tip (left) to the soma (right), as indicated by the arrows. Vertical axis: arbitrary units of fluorescence intensity. Inset: mEGFP-channel images of the process used for the signal intensity scan. Scale bar, 1 μm. (b) Left: Process tip localization of the transporters, calculated as ratios of the average signal intensity within 0.5 μm from a tip and the average signal intensity of the following 0.5 μm of representative processes. Right: Intensity of process shaft localization of the transporters were calculated as ratios of the average signal intensities within 1 μm length of a process next to the stem, and the following 1 μm length within the stem, normalized by the signal intensity of Lck-mCherry. 28–30 cells for each construct were analysed in a blinded manner. Data are presented as mean ± s.e.m., with p values of Tukey-Kramer test.

Next, to study glutamate transporter localization within finely branched astrocytic processes that are surrounded by neurons and glia, we applied adenoviruses encoding Lck-mCherry or mEGFP labelled transporters to hippocampal organotypic slice cultures. The viruses selectively transfected astrocytes, which possessed the characteristic finely branched cellular morphologies. Density of full-length mEGFP-EAAT2 and mEGFP-ASCT1 were higher at the processes compared to the stem branches by at least 1.4 fold (Fig. 4). Both mEGFP-EAAT2 and mEGFP-EAAT2ΔNC had higher density at process tips. In contrast, the signal intensity of mEGFP-EAAT1 was lower at processes than at stems by 0.7 fold, indicating primarily localization inside the stems, reflecting its localization in the ER. Removal of cytoplasmic tails of EAAT1 slightly enhanced the localization of the transporter to processes, though the enhancement did not reach the statistical significance. In contrast, the ASCT1ΔNC transporter core, which localized to filopodia tips in primary culture astrocytes, failed to enter fine astrocytic processes in hippocampal slices, with less than 0.5 fold density than the stem. These results indicate that interactions between astrocytes and their surrounding cells influence the localization of the SLC1 transporters in a subtype-specific manner.

## Discussion

Our studies indicate that the function of SLC1 transporter domain is not limited to transporting substrates, and it acts as a sorting signal to process tips. Immune-electron microscopy studies indicate that the two major glutamate transporters, EAAT2 and EAAT1, are primarily localized on surface of astrocytes, concentrated at interface with neurons^11^,^12^,^13^,^14^,^15^. Localization at process tips can potentially bring the transporters close to glutamate release sites near the site of contact with neurons, where the transporters are most needed. Removing glutamate at the site of release will reduce the overflow of glutamate from synaptic clefts. The efficiency of glutamate removal is critical for neuronal function, because activation of extrasynaptic glutamate receptor is neurotoxic and promotes neuronal cell death^31^. How far glutamate released at nerve terminus reaches affects functioning of the nervous system. For example, expression level of neuronal glutamate transporter EAAT4 in Purkinje cells of cerebellum affects extrasynaptic neuroglial signalling^32^. Reduced process tip localization of C186S mutant of EAAT1, which causes episodic ataxia, underscores the importance of this property for proper functioning of the nervous system.

In addition to these astrocytic glutamate transporters, there are some neuronal glutamate transporters. Among these, EAAT4 is primarily expressed in Purkinje cells of cerebellum, where it is enriched in of spines or dendrites facing astrocytes^33^. Another neuronal glutamate transporter EAAT3/EAAC1 is broadly localized to dendritic shafts and filopodia in young neurons, but it forms clusters in spines or dendritic filopodia in older neurons^34^. These reports indicate that the property of their transmembrane transporter domains as a filopodia tip localizer also contributed to the process localization in neurons.

Another trimeric SLC1 transporter family member, a neutral amino acid transporter ASCT1 also showed filopodia localization both as an intact protein or as a transmembrane transporter domain in astrocytes of primary culture or in COS-7 cells. However, intact ASCT1 did not show robust localization to astrocyte processes in cultured hippocampus slices, and its transmembrane transporter domain failed to be at the process tips. From these results, it emerges that while it is a common property of SLC1 transporters to be at the process tips, the process tip localization of ASCT1 is more dependent on extracellular substrates or on availability of surface for attachment. Despite of the lack of process tip localization of ASCT1 in astrocytes of hippocampal slice culture, an immune-electron microscopy study indicates that ASCT1 is also localized to the surface of astrocytes facing neurons^35^. This difference may be due to physiological properties of astrocytes in slice cultures. Many neurons and astrocytes are damaged during slice preparation and intimate cell-cell contacts in intact brains may be lost as a result. In addition, although astrocytes of organotypic slice cultures keep the finely branched structure and are able to support neuronal plasticity, many of them respond to the damage by gaining properties of reactive astrocytes. As a result, extracellular substrates or cell-cell contacts, which supported the process tip localization in COS-7 cells or in intact brains, may be lost in cultured slices. The differences between the glutamate transporters and neutral amino acid transporters indicate that the filopodia tip localization is regulated in a subtype specific manner.

SLC1 transporters within the process tips have a potential to serve as foci for recruitment of proteins required at astrocyte-neuron interface. Both EAAT1 and EAAT2 co-immunoprecipitate with Na^+^/K^+^ ATPase, which maintains the electrochemical gradient of Na^+^ for glutamate transporters at a cost of ATP, or glycolytic enzymes or mitochondrial proteins for supplying ATP for the Na^+^/K^+^ ATPase^36^,^37^,^38^. By detecting and transporting extracellular glutamate, glutamate transporters in the astrocyte processes can potentially act as sensors of neuronal synapses, and once astrocyte processes recognize neurons, the processes will be stabilized at a site of contact and start recruiting and assembling astrocytic proteins through interactions with scaffolding proteins.

Our studies of using deletion mutants of EAAT2 show that, intra-bowl insertions at the centre of the trimers are required for their filopodia tip localization. In addition to mammalian SLC1 transporters, other metazoan SLC1 transporters, such as *Drosophila* or *C. elegans*, also have intra-bowl insertions. In contrast, SLC1 transporters of single cell organisms, including fungi, archaea and bacteria do not have the intra-bowl insertion. Accordingly, GluTPh, an archaea glutamate transporter homolog without an intra-bowl insertion did not localize to filopodia tips. The difference between SLC1 transporters of metazoans and single cell organisms is reasonable because it is better for multicellular organisms with the nervous systems to have a transporter with a property to localize to the tips of cellular protrusions. We consider intra-bowl insertions are functional as a part of the transporter domain and it must cooperate with other parts of transporter, indicated by the lack of filopodia tip localization of a chimera construct containing transmembrane regions of GluTPh and intra-bowl insertion of EAAT2. Differential effect of glycosylation removal on the localization within filopodia suggests that multiple molecules may contribute to the filopodia localization. For example, extracellular lectins may interact with N-linked glycans to stabilize transporters through the length of filopodia, while other extracellular matrix molecule with peptide motif binding domains, such as immunoglobulin-like domains, may interact with the intra-bowl insertion at the process tips.

## Methods

All procedures related to the care and treatment of animals are performed in accordance with Japanese national guidelines and regulations, and were approved by the animal resource committee of the School of Medicine, Keio University.

### Constructs

Mouse EAAT1 and EAAT2 were cloned using PCR from a mouse brain cDNA library, a human ASCT1 cDNA clone was purchased from the Kazusa DNA Research Institute Collection, and GluTPh was cloned using PCR from *Pyrococcus horikoshii* genomic DNA purchased from the Biological Resource Center of National Institute of Technology and Evaluation. Deletion constructs containing the following amino acid residues: i) EAAT1 ΔNC: 45–506, ii) EAAT1 ΔN: 45-C-term, iii) EAAT2 ΔNC: 41–504, iv) EAAT2 ΔI: N term-187 and 240-C term, v) ASCT1 ΔNC: 39–486, vi) GluTPh: 7-C terminus, vii) GluTPh-IBI EAAT2: GluTPh 7–142, EAAT2 179–243, GluTPh 153-C term were inserted into pmEGFP-C1 or pmCherry-C1, which are derived from pEGFP-C1 (Clontech) by introducing A206K mutation to monomerise the EGFP^39^. EAAT1 with C-terminally tagged mEGFP, was constructed by insertion of EAAT1 to pmEGFP-N1 derived from pEGFP-N1 (Clontech). EAAT1 and EAAT1ΔN without tags were constructed by replacing mEGFP with a start codon of mEGFP-tagged constructs. Glycosylation site mutant of EAAT2 (EAAT2 ΔG) has double mutations of N205Q and N215Q.

### Recombinant Adenoviruses

Recombinant adenoviruses were made using the Gateway system (Invitrogen). Constructs to be expressed were inserted into pENTR11-dual vector and then transferred to pAv/CMV/DEST-V5 adenoviral vectors. The adenoviral vectors were introduced to HEK293A cells (Invitrogen) using Lipofectamine LTX and Lipofectamine plus (Invitrogen). Recombinant viruses were amplified by transfection of HEK293A cells grown in 16 cm plates, and harvested 3 days after transfection. The cells were harvested by centrifugation and re-suspended in 2 ml hippocampal slice culture medium, and lysed by 3 cycles of freezing and thawing, followed by centrifuge at 600 × g. The resulting virus suspensions obtained as the supernatant contained 10^8^–10^9^ pfu/μl of adenovirus against HEK293A cells.

### Cultured cell lines

COS-7 cells were plated in 24-well plates containing uncoated glass coverslips at 5 × 10^4^ cells/well in DMEM/10% FBS, and the indicated plasmids were transfected using Lipofectamine LTX and Lipofectamine Plus (Invitrogen) or linear polyethyleneimine (Polysciences) on the day of plating. The transfected cells were imaged following 2 days of incubation.

### Astrocyte primary culture

Primary cultures of mouse astrocytes were prepared from C57BL/6JJ P0 mice as described previously^40^. On DIV 14–21, the astrocytes are seeded in 24-well plates containing uncoated glass coverslips at 1 × 10^5^ cells/well. Recombinant adenoviruses are added 4–7 days after plating at ~1000 m.o.i/well, and the astrocytes were imaged on the next day of infection.

### Hippocampal organotypic slice cultures

Hippocampal organotypic slice cultures were prepared from P7 SD rats as previously described, using “donut” plates containing Millipore membranes^41^. The membranes with slices at DIV 5–10 were removed from the donut plates and incubated slice-side-down on a 50 μl droplet of the adenovirus solution at 37 degrees for 30 min, and returned to the original plate for an additional 2-day incubation.

### Confocal microscope imaging

The coverslips with the COS-7 cells or the primary cultured astrocytes were transferred to a glass-bottomed culture plates filled with HBSS^42^ for confocal imaging. Imaging of hippocampal organotypic slice cultures was carried out in a circulating solution containing (mM): NaCl 119; KCl 2.5; CaCl_2_ 4; MgCl_2_ 4; NaHCO_3_ 26.2; NaH_2_PO_4_ 1 and glucose 11, aerated with 95% O_2_ and 5% CO_2_. Images were taken with an FV BX61W1 laser scanning confocal microscope system (Olympus) with LUMPlanFL IR 0.90w 60 × objective lens (Olympus), Fluoview software, with excitation at 488 nm for monomeric EGFP (mEGFP) (Zacharias et al., 2002) and 559 nm for mCherry. Signal intensities were analysed using Metamorph (Molecular Devices) or ImageJ^43^ softwares in a blinded manner. Anti-GLAST antibody raised against C terminus of EAAT1 (GLAST-Rb-Af660, Frontier Institute) was used for immunofluorescence.

## Author Contributions

M.K.H. and M.Y. designed the research and wrote the manuscript, M.K.H. performed experiments and analysed the data.
